# Outcomes Following On-Pump Versus Of-Pump CABG: Apprising the “Bypassed”

**DOI:** 10.21470/1678-9741-2022-0088

**Published:** 2023

**Authors:** Rohan Magoon, Iti Shri, Devishree Das

**Affiliations:** 1 Department of Cardiac Anaesthesia, Atal Bihari Vajpayee Institute of Medical Sciences (ABVIMS) and Dr. Ram Manohar Lohia Hospital, New Delhi, India.; 2 Department of Cardiac Anaesthesia, Cardiothoracic Centre, CNC, All India Institute of Medical Sciences, Ansari Nagar, New Delhi, India.

Dear Editor,

The Rösler et al.^[^1^]^ diligent propensity-matched analysis and retrospective comparison of outcomes in a large Southern Brazilian cohort undergoing on-pump and of-pump coronary artery bypass grafting (CABG) is undeniably a noteworthy addition to an ardently researched domain. Nonetheless, we wish to highlight certain caveats in the larger context of the research subject. Firstly, we miss the account on the comparison of renal outcomes in the Rösler et al.^[^1^]^ evaluation. Notably, a systematic review and meta-analysis by Cheungpasitporn et al.^[^2^]^ involving 33 studies and 17,322 patients revealed a significantly lower incidence of acute kidney injury (AKI) in the of-pump CABG group compared to the on-pump group (19.1% vs 22.2%, respectively). The researchers hence concluded a protective role of of-pump CABG on the incidence of AKI (pooled risk ratio: 0.87; 95% confidence interval [CI]: 0.77–0.98; I2=5%). Although a few researchers do not suggest a statistically significant reduction in the incidence rate of AKI between of-pump and on-pump CABG, they adequately emphasize the prognostic ramifications of the index complication^[^3^]^.

Secondly, given postoperative stroke was an important outcome under consideration in the analysis of Rösler et al.^[^1^]^, the absence of intergroup matching for the prevalence of carotid artery stenosis as well as incidence of postoperative atrial fibrillation (POAF) is difficult to overlook. On one end, carotid atherosclerosis is strongly correlated with coronary artery disease patients scheduled for CABG, lending a complication rate of 2% to 18%^[^4^]^. On the other end, POAF has also been described to occur with an incidence of 15-36% after CABG by Kerwin et al.^[^5^]^, who simultaneously suggest an accentuated risk of stroke owing to POAF (adjusted odds ratio: 1.88; 95% CI: 1.02-3.46; *P*-value=0.04) in their meta-analysis including 19 studies and 1,29,628 patients.

Thirdly, it remains of additional interest if need of repeat revascularization was within the purview of the major adverse cardiovascular and cerebrovascular events (MACCE) definition employed by Rösler et al.^[^1^]^. The importance of the former is heralded in the study findings of Rupprecht et al.^[^6^]^, who reported 10.2-25% 30-day mortality rate owing to the need of repeat revascularization following primary CABG. Indeed, Rösler et al.^[^1^]^ also focused on the 30-day mortality in their analysis. At the same time, the recent systematic review by Bosco et al. elucidates a considerable heterogeneity prevailing in literature for MACCE composite end points conferring a challenge in comparing and interpreting results across observational studies^[^7^]^. Therefore, presentation of a formal MACCE definition in the Rösler et al.^[^1^]^ methodology could have enhanced the lucidity of their study.

Lastly, the description of the corresponding transfusion requirements in the on-pump and of-pump groups would have further consolidated the comparative perspective of the study by Rösler et al.^[^1^]^. The aforementioned becomes particularly relevant when transfusion cognates poor outcomes in CABG, as delineated by Kuduvalli et al.^[^8^]^. While the authors have made a conscientious endeavour in comparing outcomes after on-pump *vs.* of-pump CABG, apprising the “bypassed” parameters can be pivotal to a sound comprehension of the outcome predisposition following “bypass” surgery.
